# Short-term costs of conventional *vs* laparoscopic assisted surgery in patients with colorectal cancer (MRC CLASICC trial)

**DOI:** 10.1038/sj.bjc.6603203

**Published:** 2006-06-06

**Authors:** P J Franks, N Bosanquet, H Thorpe, J M Brown, J Copeland, A M H Smith, P Quirke, P J Guillou

**Affiliations:** 1 Centre for Research & Implementation of Clinical Practice, Thames Valley University, 32–38 Uxbridge Road, London, W5 2BS, UK; 2 Department of Bio-engineering, Imperial College, Exhibition Road, London SW7 2BX, UK; 3 Clinical Trials Research Unit, Leeds Institute of Molecular Medicine, Epidemiology and Cancer Research, University of Leeds, Leeds, UK; 4 Academic Unit of Surgery, St James's University Hospital, Leeds, UK; 5 Academic Unit of Pathology, Leeds Institute of Molecular Medicine, Epidemiology and Cancer Research, University of Leeds, Leeds, UK

**Keywords:** colorectal cancer, surgery, laparoscopic, cost analysis

## Abstract

The short-term clinical results of the CLASICC trial indicated that clinical outcomes were similar between laparoscopic and open approaches. This study presents the short-term (3 month) cost analysis undertaken on a subset of patients entered into the CLASICC trial (682 of 794 patients). As expected the costs associated with the operation were higher in the 452 patients randomised to laparoscopic surgery (lap) compared with the 230 randomised to open procedure (open), £1703 *vs* £1386. This was partially offset by the other hospital (nontheatre) costs, which were lower in the lap group (£2930 *vs* £3176). The average cost to individuals for reoperations was higher in the lap group (£762 *vs* £553). Overall costs were slightly higher in the lap group (£6899 *vs* £6631), with mean difference of £268 (95%CI −689 to 1457). Sensitivity analysis made little difference to these results. The cost of rectal surgery was higher than for colon, for lap (£8259 *vs* £5586) and open procedures (£7820 *vs* £5503). The short-term cost analysis for the CLASICC trial indicates that the costs of either laparoscopic or open procedure were similar, lap surgery costing marginally more on average than open surgery.

The last 20 years have seen major improvements in survival following colorectal cancer as a consequence of advances in early diagnosis, improved chemotherapy and radiotherapy and advances in surgical technique (Hayne *et al*, 2001). The application of laparoscopic surgery to colorectal disease started in the early 1990s, and was instigated as a means of offering patients a more rapid recovery, fewer complications and shorter hospital stay (Jacobs *et al*, 1991). Conversely, issues have arisen with regard to the high proportion of patients who require a conversion to an open procedure, and the generally longer theatre time required with costs associated with them. However, despite advocates of this technique, few have been subjected to randomised controlled clinical trials of therapy (Stage *et al*, 1997; Hewett *et al*, 1998; Lacy *et al*, 2002). Nonrandomised studies have examined cost, some of which have indicated lower costs with laparoscopic surgery (Bokey *et al*, 1996; Philipson *et al*, 1997), while others have found the results to be equivocal (Falk *et al*, 1993; Bergamaschi and Arnaud, 1997; Joo *et al*, 1998; Khalili *et al*, 1998; Young-Fadok *et al*, 2001).

The Conventional *vs* Laparoscopic Surgery in Colorectal Cancer (CLASICC) trial, is a UK multicentre pragmatic trial, which randomised patients between July 1996 and July 2002 to either laparoscopic surgery or an open procedure. The trial was designed to randomise patients to laparoscopic or open surgery (Stead *et al*, 2000). Details of the overall trial design and short-term clinical outcomes are reported elsewhere (Guillou *et al*, 2005). In brief, the trial was designed to study clinical end points of survival and disease-free interval. A total of 794 patients from 27 centres (32 surgeons) provided informed consent and were randomised to laparoscopic (526 patients) or open surgery (268 patients) in a ratio of 2 : 1, with stratification by surgeon, proposed site of operation, presence of liver metastases and preoperative radiotherapy administration. Patients with both colon (413 patients) and rectal (381 patients) cancer were included in this trial.

The shortterm end points of this trial indicated similar results between the randomised groups in respect of positive resection margins, proportion of patients with Dukes' C tumours and in-hospital mortality (Guillou *et al*, 2005). Secondary short-term end points of surgical complication rates (intraoperatively and up to 30 days and 3 months postoperatively), transfusion requirements and quality of life up to 3 months were also similar between groups.

The purpose of the current paper is to report on the short-term cost implications of either laparoscopic or open resection for patients with colorectal cancer in the CLASICC trial.

## METHODS

The cost analysis in this trial was undertaken from the perspective of the UK National Health Service, with analysis undertaken up to three months post operation. Information on resource usage was provided at the individual patient level.

Patients for this part of the trial were included on the basis that they provided written informed consent to participate in the quality of life/health economics sub-study of the trial. This was a subset of the total patient group (*n*=682) Patients who did not give this consent, or where details of the actual operative procedure were missing at the time of analysis, were not included. Detailed theatre resource use was undertaken in a subgroup of these eligible patients, with the aim to recruit 10 patients randomised to the laparoscopic procedure and 10 patients to the open procedure from each recruiting surgeon.

### Theatre use

Theatre usage was determined on the basis of information recorded by the surgeons in the immediate postoperative period. Theatre time was calculated in the subgroup as the time between first incision to the time that the dressing was applied to the surgical wound. Theatre time for patients not in the sample was imputed from linear regressions derived from the subgroup, which related the actual operation undertaken with anaesthetic time.

Fixed theatre cost was estimated by subtracting the total variable costs (staffing and equipment costs) from the total theatre cost in the patients who underwent the standard (open) procedure. This fixed cost (£92.50) was then used with the individual patient variable cost to determine patient specific theatre cost.

Detailed records were taken of theatre staffing including surgical/anaesthetic and nursing grades present for each operation. Information from the sample was used to impute values for patients in whom this information was not available by drawing a random values from those in whom details were available, stratified by operative procedure (open or lap) and operation undertaken (open, lap or conversion). Use of disposable and nondisposable equipment was estimated by the individual surgeon's normal practice for each procedure. Requirement for blood and fluid transfusion was determined in the immediate postoperative period and for 7 days following surgery, together with blood volumes administered and type of blood used (allogenic, autologous or blood products).

### Hospital stay

Hospital stay was calculated from the date of operation to the date of discharge/death plus an additional day (preoperative admission). This hospital stay was subdivided into time on the surgical ward, time in intensive care (ICU) and time in high dependency (HDU). As this analysis relates to follow-up to 3 months, in-patient stay was truncated at 90 days.

### Complications

Intraoperative complications were recorded in the immediate postoperative period by the trial surgeon, and were subsumed within the overall theatre costs. Postoperative complications were recorded up to 30 days and 3 months postoperation. At these times information was collected on whether further surgery was undertaken. Follow-up questionnaires were sent to trial centres to provide more detailed information on reoperations, including anaesthetic time, additional in-patient stay for surgery and use of ICU and/or HDU. Postoperative complications that did not lead to further surgery were also recorded, and coded blind by a surgeon to estimate resource use. Serious postoperative complications were costed according to national figures, with account taken for in-patient stay (NHS, 2002). Minor complications were coded according to the need for antibiotics, urinary catheterisation and CT scan.

### Postdischarge health resource usage

Patients who agreed to take part in the health economics substudy of the trial were sent questionnaires about their use of health resources at both 2 weeks and 3 months postoperatively. Information was requested on the number of in-patient days, outpatient visits, general practitioner visits, use of district (community) and stoma nursing services. Details of reasons for visits were given, which allowed for exclusion of double counting of in-patient stay (operative procedure and reoperations), and outpatient visits for radiotherapy or chemotherapy, which were excluded from the analysis.

## UNIT COSTS

Unit costs were determined from a number of sources. Total hourly rate for theatre time and recovery were provided by one of the main hospitals participating, together with hotel cost for ward stay, ICU/HDU and outpatient visit (Table 1). Staffing costs were estimated as mid point of scales given in the UK literature (Netten *et al*, 2001). Cost of theatre equipment specific to the procedures undertaken were provided by a manufacturer, and given as the average selling price for the products in 2003 (personal communication). All nonspecific equipment was assumed to be incorporated into the fixed theatre costs. Reoperations were assumed to be open procedures, with variable hourly cost assumed to be identical to the mean for patients who initially underwent open surgery. Postdischarge health resource unit costs were also estimated from national published figures (Netten *et al*, 2001). Indirect costs were estimated by determining the time taken for the patients in work to return to employment, with a cutoff set to 90 days. Average salary costs of £66.24 per calendar day for those in full time employment and £33.12 per day for part time workers were taken from the Department of Work & Pensions for 2002.

Sensitivity analysis was used to challenge some of the assumptions made in the analysis, with particular respect to perioperative costs, equipment costs, recovery time, ICU cost and cost of hospital stay (ward/ICU/HDU). In this analysis a potential deviation from the assumed costs of 20% either side of the estimated costs was used to determine the robustness of the cost estimates. Analysis was also undertaken analysing patients with rectal and colon disease separately.

Average costs were determined with bootstrap estimates of confidence intervals given at 2.5 and 97.5% levels.

## RESULTS

Of the total patients included in this trial, 696 agreed to take part in the health economics substudy. A further 14 were excluded on the basis that details of the operative procedure were unknown at the time of analysis, leaving a total of 682 (452 laparoscopic, 230 open). Of these, 315 underwent a successful laparoscopic procedure (311 randomised to lap, four randomised to open), with 122 of the 437 (28%) patients requiring a conversion to open procedure. In all, 242 underwent a standard open procedure (17 randomised to lap, 225 randomised to open). A further three patients had no surgery.

Table 2 details baseline demographics for all patients included in the health economics substudy, while Table 3 presents key items of resource use for these patients. As expected, patients in the lap group had longer median duration anaesthetic time (180 *vs* 135 min), but slightly shorter median stay in hospital (10.0 *vs* 12.0 days). Intensive care was more frequently required for patients randomised to the open procedure, but the mean duration in ICU was shorter. A similar proportion of randomised patients required blood transfusion (33.6 *vs* 34.0%).

For the 233 patients included in the detailed theatre resource use subgroup, time in theatre was longer for the laparoscopic group compared with the open group (median 165 *vs* 115 min), Table 3. Theatre staffing was similar between procedures, for all grades of medical and nursing staff.

The incidence of reoperations within 3 months after surgery was similar between groups (10.6% for the lap group *vs* 9.1% for the open group), Table 4. The incidence of major complications was similar between the randomised groups (14.4% for the lap group *vs* 10.7% for the open group), as were the proportion of patients experiencing minor complications requiring antibiotics, urinary catheterisation and CT scans.

Over the 3 month postoperative period patients were seen by a wide range of health care professionals for numerous reasons. After 3 months 20.3% lap patients and 32.1% of open patients underwent a further in-patient stay (for any reason), with 75.0% lap patients and 50.8% open patients were seen as outpatients for reasons other than radiotherapy or chemotherapy. The stoma nurse saw 38.7% lap patients and 45.2% open patients (mean number of visits 4.5 *vs* 4.8). District nurses were seen by 48.7% of lap and 77.4% open patients.

### Three Month cost of health resources

Table 5 gives the overall cost of health resource use of patients who entered the health economics substudy of the CLASICC trial. The results indicate that patients randomised to laparoscopic surgery had a significantly higher cost around the perioperative period (mean difference=£317.96, 95%CI £235.39 to £395.66), however, the total cost was similar, with the laparoscopic arm having slightly higher total cost (mean difference=£268.11, 95%CI −£689.09 to £1457.52). Sensitivity analysis was used to challenge some of the assumptions made in the analysis, (Table 6). While some changes did occur with respect to the mean difference in cost, patients who underwent laparoscopic surgery had consistently higher costs associated with their procedure and follow-up. The largest difference occurred when hospital costs were challenged (£317) with smallest differences occurring with reduced equipment costs (£86).

Table 7 gives details of cost differences associated with colon and rectal cancers analysed separately. The cost of rectal surgery was higher than for colon, for both lap (£8260 *vs* £5587) and open procedures (£7820 *vs* £5503). Again the difference between randomised groups indicated a slightly higher cost for laparoscopic surgery than the open procedure, with a smaller difference for colon cancers (£83.92, 95%CI −£642.12 to £791.99) compared with rectal cancer (£439.15, 95%CI £−1293.85 to £2857.27).

Analysis was undertaken to examine the relationship between conversion rate and total cost of care. The relationship between the two was not a simple one (Table 8). Conversion rates varied between 36.1% in the second year of randomisation up to 17% in the sixth year. Despite this, the overall difference between randomised groups was greatest in the sixth year in favour of the open procedure, with years one and two (highest conversion rates), experiencing costs in favour of laparoscopic surgery. This apparent anomaly was explored by examination of actual operation performed. The cost of undertaking a successful laparoscopic procedure was highest in the sixth year (£7713.22), whereas the conversion costs were lowest (£5798.06). Lowest cost of a successful laparoscopic procedure was in year 2 (£5682.57) which also corresponded with a high cost of conversion (£8643.82).

## DISCUSSION

The CLASICC trial has a pragmatic approach to determine the outcomes of laparoscopic surgery compared with open procedures in patients with colorectal cancer. It has shown that there is a trade off in costs. The laparoscopic procedure was more expensive in terms of theatre costs, whilst the other hospital costs such as ward, ICU and HDU and complications were higher in the patients randomised to the open procedure.

The results of this trial are consistent with other costing studies of laparoscopic surgery for colorectal cancer (Philipson *et al*, 1997, Khalili *et al*, 1998, Delaney *et al*, 2003, Janson *et al*, 2004). Khalili *et al* (1998) found similar costs between treatment groups, though the operating theatre costs were higher for laparoscopic surgery. While others have found overall higher costs of the laparoscopic procedure, much of this can be explained by the results being limited to operating costs only (Philipson *et al*, 1997). Delaney *at al* used a case matched design to compare patients who underwent colorectal surgery using the laparoscopic and open procedures. Patients were matched for age, gender and disease group. Their conclusion was that laparoscopic surgery was less expensive than the open procedure despite a higher overall cost within the operating room. This was largely a consequence of shorter hospital stay (3 *vs* 6 days) and lower ward nursing, pharmacy and laboratory costs. However, of the 150 matched cases in this study only 34 laparoscopic and 47 open surgery patients were operated on for colon cancer, and information was only provided for patients while they were within the hospital system.

The recent COLOR trial has provided a similar approach to the analysis of short-term costs undertaken in the current trial, with a sample of 210 Swedish patients from a multinational trial of 1200 patients with colon cancer (Janson *et al*, 2004). Societal costs were higher in the patients who underwent conventional open surgery, although as expected operative costs were higher in patients who underwent laparoscopic surgery. The total 12-week costs were significantly higher in the laparoscopic group (difference=€2244), despite just 14 of 98 (14%) undergoing intraoperative conversion from lap to open. Their results indicated that laparoscopic surgery was not associated with shorter hospital stay, and required more reoperations. Conversely the present trial results demonstrated similar costs between groups, despite there being a higher conversion rate (28%). The present study also indicated a slightly lower duration in hospital for the patients randomised to laparoscopic surgery (13.6 *vs* 14.4 days), which would reduce costs in favour of the laparoscopic procedure.

The results show a clear difference in cost between patients who undergo colon cancer resection and those with rectal disease. The overall cost difference of undertaking laparoscopic cancer in rectal cancer was somewhat higher than for patients who underwent colon cancer resection. Despite these differences, the percentage cost difference was only around 5% of the total cost of care.

The apparent paradox in relation to the difference in costs in relation to conversion rate can be explained by observing the costs of each operation type undertaken. There was evidence that the cost of open surgery was reducing over time as a consequence of shorter hospital stay and less use of HDU. However, the cost of a successful laparoscopic operation was increasing despite a lower conversion rate. This may be a consequence of surgeons spending more time and effort in completing the operation they had originally planned, with a subsequent increase in cost for this group. Evidence to support this comes from an analysis of reoperation costs, which increased from an average of £321 in year one to £2119 in year 6.

## CONCLUSION

The analysis of cost data from 682 patients from the CLASICC trial has shown that there are similar costs involved in the laparoscopic and open procedures for colorectal cancer within the UK. On the basis that the short-term outcomes are similar, it would appear that until longer-term results for the randomised trials are made available, it would appear that both surgical options are equally acceptable in the short-term for both clinical and costs of treatment.

## Figures and Tables

**Table 1:** Key unit costs of resources used in the cost analysis

	**Unit**	**Cost (£)**	**Source**
Theatre time	Hour	577.00	A
Recovery	Hour	124.00	A
			
*Staffing*			
Consultant	Hour	45.33	B
Specialist registrar	Hour	28.49	B
Senior house officer	Hour	24.47	B
House officer	Hour	15.20	B
Nurse (F to I)	Hour	18.55	B
Nurse (D to E)	Hour	14.25	B
Nurse (A to C)	Hour	9.16	B
			
*Laparoscopic equipment*			
Scissors	Unit	75.00	C
Grasper	Unit	70.00	C
Trochar	Unit	50.00	C
Ligaclip applicator	Unit	105.00	C
Linear stapler	Unit	105.00	C
Circular stapler	Unit	225.00	C
			
*Hospital stay*			
General ward (hotel)	Day	154.00	A
HDU	Day	918.00	A
ICU	Day	1465.00	A
GP (home)	Visit	47.00	B
GP (surgery)	Visit	15.00	B
District nurse	Visit	16.00	B
Stoma nurse	Visit	16.00	B

A Leeds Hospitals NHS Trust.

B PSSRU 2001.

C Manufacturers' average selling price 2003.

**Table 2:** Demographics of patients entered into the health economics sub-study of the CLASICC Trial

	**Lap**	**Open**
*n*	452	230
*Gender*		
Male	255 (56.4%)	131 (56.9%)
Female	197 (43.6%)	99 (43.0%)
		
*Age in years*		
Mean (s.d.)	68.10 (10.48)	68.80 (11.86)
		
*WHO performance status*		
0	298 (65.9%)	135 (59.0%)
1	110 (24.3%)	71 (31.0%)
2	38 (8.4%)	22 (9.6%)
3	5 (1.1%)	1 (0.4%)
4	1 (0.2%)	0
Missing	0	1
		
*Surgery undertaken*		
Laparoscopic	311 (68.8%)	4 (1.7%)
Open	17 (3.8%)	225 (97.8%)
Conversion	122 (30.0%)	0 (0)
No operation	2 (0.4%)	1 (0.4%)
		
*Site of tumour*		
Colon	230 (50.9%)	118 (51.3%)
Rectum	222 (49.1%)	112 (48.7%)

**Table 3:** Key items of resource use

**Total group**	**Laparoscopic group**	**Open group**
*N*	452	230
Laparoscopic	311	4
Open	17	225
Conversion	122	0
No operation	2	1
		
*Duration of anaesthetic*		
Mean	183.7	139.7
Median (IQR)	180.0 (135.0–225.0)	135.0 (100.0–180.0)
Total	420	209
		
*Total length of hospital stay (days)*		
Mean	13.6	14.4
Median (IQR)	10.0 (8.0–15.0)	12.0 (9.0–15.5)
Total	424	213
		
Intensive care *n* (%)	19 (5.4%)	17 (9.9%)
	354	171
		
*For ICU stay*		
Mean	5.74	1.84
Median (ICR)	3.0 (2.0–5.0)	1.33 (1.0–2.0)
		
High Dependency n (%)	46 (14.0%)	19 (12.0%)
	328	158
		
*For HDU stay*		
Mean	2.71	3.55
Median (IQR)	2.0 (2.0–3.25)	3.0 (2.0–4.0)
		
Blood transfusion n (%)	143 (33.6%)	72 (34.0%)
	426	212
		
Allogenic blood	142 (33.3%)	72 (34.0%)
	426	212
		
Blood products	3 (0.7%)	5 (2.3%)
	426	213
		

**Subgroup**	**Laparoscopic**	**Open**
*N*	145	88
*Time in anaesthetic room*		
Mean	21.0	18.2
Median (IQR)	19.0 (14.0–25.0)	15.0 (10.0–23.0)
	143	81
		
*Time in theatre in minutes*		
Mean	172.3	118.2
Median (IQR)	165.0 (124.0–210.0)	115.0 (83.0–140.0)
	142	79

**Table 4:** Postoperative complications and use of resources

	**Laparoscopic**	**Open**
*N*		
Reoperations within 3 months	48 (10.6%)	21 (9.1%)
		
Surgical complication	34	17
Other complication	8	2
Stoma surgery	6	2
		
*Postoperative complications (excluding reoperations)*		
Major complication	62 (14.4%)	23 (10.7%)
	431	215
		
*Other complication*		
Requiring antibiotics	67 (15.5%)	38 (17.7%)
	431	215
		
*Other complication*		
Requiring urinary catheterisation	12 (2.8%)	11 (5.1%)
	431	215
		
Other complication	13 (3.0%)	5 (2.3%)
Requiring CT scan	431	215
		
**Community Health Services (up to 3 months)**		
All cause		
*GP (surgery) visits*		
Yes	202 (44.7%)	120 (52.2%)
No	250	110
Range	(1–15)	(1–7)
		
*GP (home)*		
Yes	206 (45.6%)	88 (38.3%)
No	246	142
Range	(1–10)	(1–11)
		
*Stoma nurse visits*		
Yes	175 (38.78%)	104 (45.2%)
No	277	126
Range	(1–17)	(1–17)
		
*District nurse visits*		
Yes	146 (48.7%)	178 (77.4%)
No	154	52
Range	(1–104)	(1–66)

**Table 5:** Average cost of care for randomised patients (UK pounds)

	**Laparoscopic**	**Open**		
	***n*=452**	***n*=230**		
	**Mean**	**Mean**	**Difference**	**(95%CI)**
Theatre	1703.94	1385.98	317.96	(235.39 to 395.66)
Staffing	472.47	357.93		
Equipment	512.32	331.08		
Blood	91.45	112.71		
Other	627.70	584.26		
				
Hospital costs	2930.69	3176.52	−245.83	(−678.31 to 188.07)
ITU/HDU	1030.98	1200.70		
Ward	1899.71	1975.82		
				
Chemo/radiotherapy	279.76	318.65	−38.89	(−124.35 to 36.31)
Reoperations and other complications	762.08	553.11	208.97	(−331.14 to 1087.19)
Theatre	252.35	254.54		
ITU/HDU	372.73	0.0		
Ward	88.58	266.49		
Complications	48.41	32.08		
				
Community health	190.48	203.44	−12.96	(−54.56 to 1.05)
				
Indirect costs	1032.66	993.81	38.85	(−305.82 to 389.35)
				
Total	6899.63	6631.52	268.11	(−689.09 to 1457.52)

Confidence intervals generated by bootstrap method (1000) iterations, and given for 2.5% and 97.5% percentiles.

**Table 6:** Sensitivity analysis

	**Cost difference (Lap-open)**	**95%CI**
Base case	268.11	−689.09 to 1457.52
*Perioperative costs*		
−20%	204.52	−701.10 to 1439.96
+20%	331.71	−659.03 to 1465.03
		
*Equipment costs*		
−20%	86.87	−817.58 to 1364.69
+20%	218.95	−764.58 to 1477.99
		
*Recovery cost*		
−20%	266.55	−597.54 to 1487.66
+20%	269.67	−662.85 to 1456.99
		
*ICU cost*		
−20%	237.54	−641.61 to 1364.71
+20%	298.68	−638.13 to 1530.53
		
*Hospital costs (Ward, ITU, HDU)*		
−20%	317.27	−593.31 to 1427.99
+20%	218.95	−840.25 to 1504.95

**Table 7:** Average cost of care for colon and rectal cancer patients (UK pounds)

	**Laparoscopic**	**Open**		
	***n*=230**	***n*=118**		
	**Mean**	**Mean**	**Difference**	**(95% CI)**
*Colon*				
Theatre	1596.14	1327.41	268.73	(172.91 to 371.01)
Hospital	2517.22	2666.98	−149.76	(−594.34 to 316.56)
Chemo/radiotherapy	185.41	174.49	10.92	(−70.58 to 84.26)
Reoperations/	192.22	397.36	−205.14	(−464.25 to 31.58)
Other complications				
Community services	149.54	162.24	−12.70	(−57.10 to 8.07)
Indirect costs	946.39	774.52	171.87	(−277.54 to 590.89)
Total	5586.93	5503.01	83.92	(−642.12 to 791.99)
				

	**Laparoscopic**	**Open**		
	***n*=222**	***n*=112**		
	**Mean**	**Mean**	**Difference**	**(95% CI)**
*Rectum*				
Theatre	1815.64	1447.69	367.95	(243.54 to 483.57)
Hospital	3359.07	3713.37	−354.30	(−1131.86 to 366.54)
Chemo/radiotherapy	377.51	470.53	−93.02	(−228.04 to 41.38)
Reoperations/	1352.48	717.21	635.27	(351.21 to 2297.64)
Other complications				
Community services	232.88	246.83	−13.95	(−76.66 to 38.83)
Indirect costs	1112.04	1224.84	−102.80	(−576.04 to 367.67)
Total	8259.64	7820.49	439.15	(−1293.85 to 2857.27)

Confidence intervals generated by bootstrap method (1000) iterations, and given for 2.5 and 97.5% percentiles.

**Table 8:** Average cost of care for randomised groups and actual operation performed according to year of trial entry

**Year**	**Conversion rate (%)**	**Laparoscopic Mean**	**Open mean**	**Difference**	**(95% CI)**
*Randomised groups*					
1	33.3	6850.49	7009.81	–159.32	(−1806.17 to 1500.76)
2	36.1	6711.46	7041.89	−330.43	(−2095.52 to 1551.85)
3	28.6	6523.52	6447.73	75.79	(−1428.62 to 1535.70)
4	29.0	7098.15	6845.17	252.98	(−1522.74 to 1946.65)
5	29.6	6847.73	6362.10	485.63	(−1172.42 to 2233.04)
6	17.0	7309.22	6268.21	1041.01	(−1790.83 to 5232.97)
					

	**Conversion rate (%)**	**Successful lap mean**	**Conversion mean**	**Open mean**	
*Actual operation performed*					
1	33.3	6151.12	7623.27	7365.22	
2	36.1	5682.57	8643.82	6939.80	
3	28.6	5942.37	7876.73	6496.02	
4	29.0	6339.70	9238.64	6656.42	
5	29.6	6296.47	8380.52	6238.77	
6	17.0	7713.22	5798.06	6167.66	

Confidence intervals generated by bootstrap method (1000) iterations, and given for 2.5% and 97.5% percentiles.

## Appendix: Contributors to the Trial

*Participating Institutions and Surgeons*

Airedale General Hospital, Keighley – K Kapadia, R Khan;

Bedford General Hospital – R Foley;

Bristol Royal Infirmary – M Thomas;

Castle Hill Hospital, Hull – J Monson, C Duthie;

Colchester General Hospital-R Motson;

Darent Valley Hospital, Dartford – M Parker;

Edinburgh Royal Infirmary – D Bartolo;

Freeman Hospital – A Horgan;

Leeds General Hospital – P Sagar;

Leicester General Hospital – W Barrie;

Mayday University Hospital – R Swift;

Medway Maritime Hospital – H Westapel;

Ninewells Hospital – K Campbell;

Prince Charles Hospital – P Haray;

Princess Elizabeth Hospital, Guernsey – M Van Den Bossche;

Queens Medical Centre – J Scholefield;

Royal Gwent Hospital – K Vellacott;

Royal Liverpool Hospital – M Hershman;

Royal United Hospital – J Tate;

Royal Victoria Infirmary – J Varma, H Gallagher;

St James's University Hospital – P Guillou, A Windsor;

St Peter's Hospital – H Scott;

Stepping Hill Hospital – G Deans;

University Hospital of Wales – B Rees, D Carey;

Whipps Cross Hospital – j Wellwood;

William Harvey Hospital – N Taffinder;

Yeovil District Hospital – R Kennedy.

*Local Nominated Pathologists*

Airedale General Hospital – P Da Costa, J O'Dowd;

Bedford General Hospital – D Rimmer;

Colchester General Hospital – P Conn;

Darent Valley Hospital, Dartford – P Thebe;

Edinburgh Royal Infirmary – H Gilmour;

Hull Royal Infirmary – A MacDonald;

John Radcliffe Infirmary – B Warren;

Leeds General Hospital – P Quirke;

Leicester General Hospital – E MacKay;

Mayday University Hospital – A Arnaout;

Medway Maritime Hospital – R Lindley;

Ninewells Hospital – F Carey;

Prince Charles Hospital – S Kiberu;

Princess Elizabeth Hospital, Guernsey – C Chinyama;

Queens Medical Centre – D Jenkins;

Royal Liverpool Hospital – J Nash;

Royal United Hospital-N Rooney;

Royal Victoria Infirmary – J Shrimanker;

St James's University Hospital-N Scott, J Wyatt;

St Peter's Hospital – N Ratcliffe;

Stepping Hill Hospital – R Hale;

University Hospital of Wales – G Williams;

William Harvey Hospital – A Abdulkadir;

Yeovil District Hospital – J Sheffield.
